# Magnetic Microdimer as Mobile Meter for Measuring Plasma Glucose and Lipids

**DOI:** 10.3389/fbioe.2021.779632

**Published:** 2021-11-26

**Authors:** Shimin Yu, Zhongqi Sun, Zhanxiang Zhang, Haoran Sun, Lina Liu, Wuyi Wang, Mu Li, Qingsong Zhao, Tianlong Li

**Affiliations:** ^1^ Department of Pharmacy, The Second Affiliated Hospital of Harbin Medical University, Harbin, China; ^2^ State Key Laboratory of Robotics and System, Harbin Institute of Technology, Harbin, China; ^3^ Department of Radiology, The Second Affiliated Hospital of Harbin Medical University, Harbin, China; ^4^ Chongqing Research Institute of HIT, Harbin, China; ^5^ Department of Endocrinology, The Fourth Affiliated Hospital of Harbin Medical University, Harbin, China

**Keywords:** magnetic microdimer, point-of-care testing, angle of procession, peak velocity, plasma glucose, plasma lipids

## Abstract

With the development of designed materials and structures, a wide array of micro/nanomachines with versatile functionalities are employed for specific sensing applications. Here, we demonstrated a magnetic propelled microdimer-based point-of-care testing system, which can be used to provide the real-time data of plasma glucose and lipids relying on the motion feedback of mechanical properties. On-demand and programmable speed and direction of the microdimers can be achieved with the judicious adjustment of the external magnetic field, while their velocity and instantaneous postures provide estimation of glucose, cholesterol, and triglycerides concentrations with high temporal accuracy. Numerical simulations reveal the relationship between motility performance and surrounding liquid properties. Such technology presents a point-of-care testing (POCT) approach to adapt to biofluid measurement, which advances the development of microrobotic system in biomedical fields.

## Introduction

Redundant glucose and lipids (cholesterol and triglycerides) in human blood can cause severe health problems such as diabetes mellitus, heart disease, arteriosclerosis, hypertension, hyperlipidemia, and cerebral thrombosis (Wang and Hu, 2020). Glucose, cholesterol, and triglycerides plasma levels have been important parameters for assessing risk factors of a population in disease diagnosis. With the growing demands for quick and convenient on-site diagnostic information, simple and fast blood glucose and blood lipid determination is essential and beneficial for early diagnosis. This goal can be pursued using point-of-care testing (POCT) (Sautter et al., 2018). Some POCT strategies and instruments that can measure glucose, cholesterol, and triglyceride levels in the blood have been developed (Rapi et al., 2009; Freckmann et al., 2014; Ning et al., 2021). These methods are quickly and highly reliable, but require extensive sample, advanced instruments, and qualified personnel operation. The past decade has witnessed a boom in the development of micro/nanomachines, which would open up an avenue and offer extensive opportunities for efficient sensing systems with excellent selectivity and sensitivity (Wang et al., 2012; Li J. et al., 2017).

Currently, plenty of micro/nanomachines get power from surrounding chemical fuel (Gao et al., 2011; Ma et al., 2016; Zhuang et al., 2017) or by harnessing external energy sources, such as magnetic (Li T. et al., 2017; Xie et al., 2019; Alapan et al., 2020; Fan et al., 2020; Wang et al., 2021), light (Dong et al., 2016; Palagi et al., 2016; O’Neel-Judy et al., 2018; Wang et al., 2020), ultrasound (Wu et al., 2014; Xu et al., 2015; Ren et al., 2019), and electric (Demirörs et al., 2017; Alapan et al., 2019), and these combinations have been developed. Particularly, due to excellent maneuverability and high precision at the nano- and microscale, magnetically propelled micro and nanoswimmers provide great potential in targeted drug delivery (Felfoul et al., 2016; Gao et al., 2016; Xu H.et al., 2018), cell manipulation (Chang et al., 2019; Liao et al., 2019; Wang et al., 2020), minimally invasive surgery (Gao et al., 2015; Chen and Wang, 2020), guided imaging (Wu et al., 2014; Nothnagel et al., 2016; Medina-Sánchez et al., 2017), environmental remediation (Dekanovsky et al., 2020; Sun et al., 2020), and sensing (Moo et al., 2014; Ezhilan et al., 2015). More specifically, the enhanced physicochemical properties and motility of actively artificial nanomachine results in greater binding efficiency and synergic effect of micro/nanoscale dimensions and active motion, which can be useful in physical and chemical sensing (Yu et al., 2019; Ji et al., 2021). Three main applications of using magnetically driven micro/nanomotors for sensing and biosensing are investigated: 1) detection of the local microenvironment properties (e.g., fluid viscosity, ion strength) based on movement parameters (e.g., velocity, wobbling angle) of magnetic propelled microswimmers (Zhang et al., 2019), 2) acting as signal amplifier to enhance the detection sensitivity and efficiency for tagged signals (e.g., fluorescence) (Zhang et al., 2019), and 3) guiding and transporting payloads (i.e., biotin and avidin) to a user-defined site under the steering of external magnetic field (Park and Yossifon, 2020). To date, a mass of maneuvering micro/nanomachines with different morphologies, including tubular (Xu B. et al., 2018), Janus spherical (Li et al., 2018; Pourrahimi et al., 2018), wheel-like (Yang et al., 2019), body-deformable (Li et al., 2016), and helical shape (Ghosh et al., 2018; Wang et al., 2018), have been fabricated as mobile sensors capable of detecting analytical signals in real time directly or decorated with different receptors on their surface. The introduction of motion dimension further increases the accuracy of the various sensing abilities at local sites in 3D. However, there are no reports regarding microrobot-based microdevice for plasma glucose and lipid measurement. Hence, there is a current need in biomedical demands for new cost-effective, real-time, and easily integrated system for measuring plasma glucose and lipids.

In this work, a magnetically actuated microdimer was first used to estimate the concentration of plasma glucose and plasma lipid. The inconsistency in the length and width of geometric structure leads to changing posture with increase in frequency, presenting three motion modes of tumbling, wobbling, and rolling, while accompanying varying motion speeds. A similar tendency happens when the viscosity varies appreciably with the level of glucose and lipid of the plasma, which guides the detection of glucose, cholesterol, and triglycerides in plasma using the motility performances of velocity and posture. The method of manipulation with high spatial and temporal accuracy-based noninvasive magnetic actuation can be applied in the detection of most types of fluid samples.

## Materials and Methods

### Synthesis of Magnetic Janus Microspheres

The silica microparticles with a diameter of 8 μm (Aladdin, China) was used as the base particles to prepare the magnetic Janus microspheres. Primarily, the silica microspheres were washed three times with deionized water and redispersed in ethyl alcohol. Then the sample was spread onto glass slides and dried uniformly to form microsphere monolayers. The microspheres were sputter coated with a 100-nm nickel layer using an ion-sputtering apparatus at 90° angle of incidence. After the fabrication, the sputtered microparticles were released from the substrate using a bath sonicator in ethanol and dispersed in ultrapure water. All magnetic microspheres were stored in ultrapure water at room temperature when not in use.

### Magnetic Experiments

The controllable magnetically actuated locomotion of microdimer was driven by homogeneous rotating magnetic field generated by a vision-based magnetic navigation system. The external rotating uniform magnetic field consists of a three degrees of freedom Helmholtz coil system, a multifunction data acquisition, and a three single-channel output power amplifier. Various required rotating magnetic fields in any plane of 3D space were achieved by controlling the current and the voltage of Helmholtz coils.

### Optical Observation and Tracking

In order to achieve real-time observation of swimmers, the external magnetic field setup was placed on the observation platform of an inverted microscope (IX73, Olympus, Japan) to achieve real-time observation of microdimers. Locomotion videos of Janus microdimers were captured by a digital CCD camera (DP74, Olympus, Japan) and analyzed by using ImageJ to obtain the trajectories, velocities, and postures of swimmers.

## Results and discussion

### Point-of-care-testing System for Clinical Index

**FIGURE 1:**
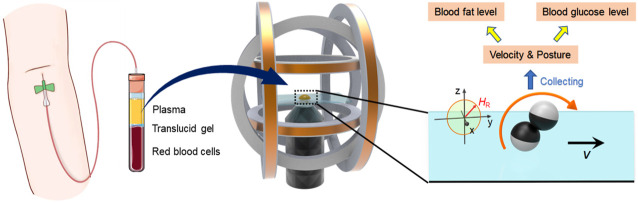
Conceptual schematic illustration of the estimation of plasma glucose and lipids using magnetic propelled microdimers.

As routine clinical parameters, glucose and lipid level detection have become the main approaches to prevent various human diseases such as diabetes, hypertension, and other cardiovascular diseases. To meet the demand of affordable and efficient clinical diagnostics, POCT has gained more and more attention and become in full swing for glucose, cholesterol, and triglyceride detection. Figure 1 schematically shows the detection strategy using a microdimer under the external magnet. Whole blood was divided into three layers of red blood cells, translucid gel, and plasma. After centrifuging for 3 min at 3,000 rpm, the upper layer of the plasma was taken out and used for the experiments. The plasma containing Janus microspheres was placed into the center of a triaxial Helmholtz coil system (two x coils, two y coils, and two z coil) fixed on an inverted microscope. The microdimers were actuated using a rotating uniform magnetic field with strength of 5 mT and frequency of 1–30 Hz. The rolling direction of the microdimer was controlled by changing the orientation of applied magnetic field. Motion speed and posture in the plasma with different glucose, cholesterol, and triglyceride concentrations were captured and analyzed to seek the estimation approach for glucose and lipid levels.

### Propulsion of Magnetic Microdimers on Flat Surfaces

In the method described here, artificially prepared plasma samples with different viscosities were prepared using different glucose (or cholesterol and triglyceride) concentration aqueous solutions. We could maneuver and position microdimers with a micrometer-scale resolution using externally applied magnetic fields, while simultaneously imaging its position and orientation to estimate the glucose, cholesterol, and triglyceride concentration in real time. The flat surface is critically important in converting the magnetically induced rotation to linear translation. The micro and nanorobots based on this “surface-assisted locomotion” mechanism is called “surface walkers.” Such time-reversible reciprocal locomotion can still generate an effective propulsion in the blood (non-Newtonian fluids). In addition, propulsion using rotating magnetic fields is noninvasive, and the microdimers are stable in other types of biological fluids.

**FIGURE 2:**
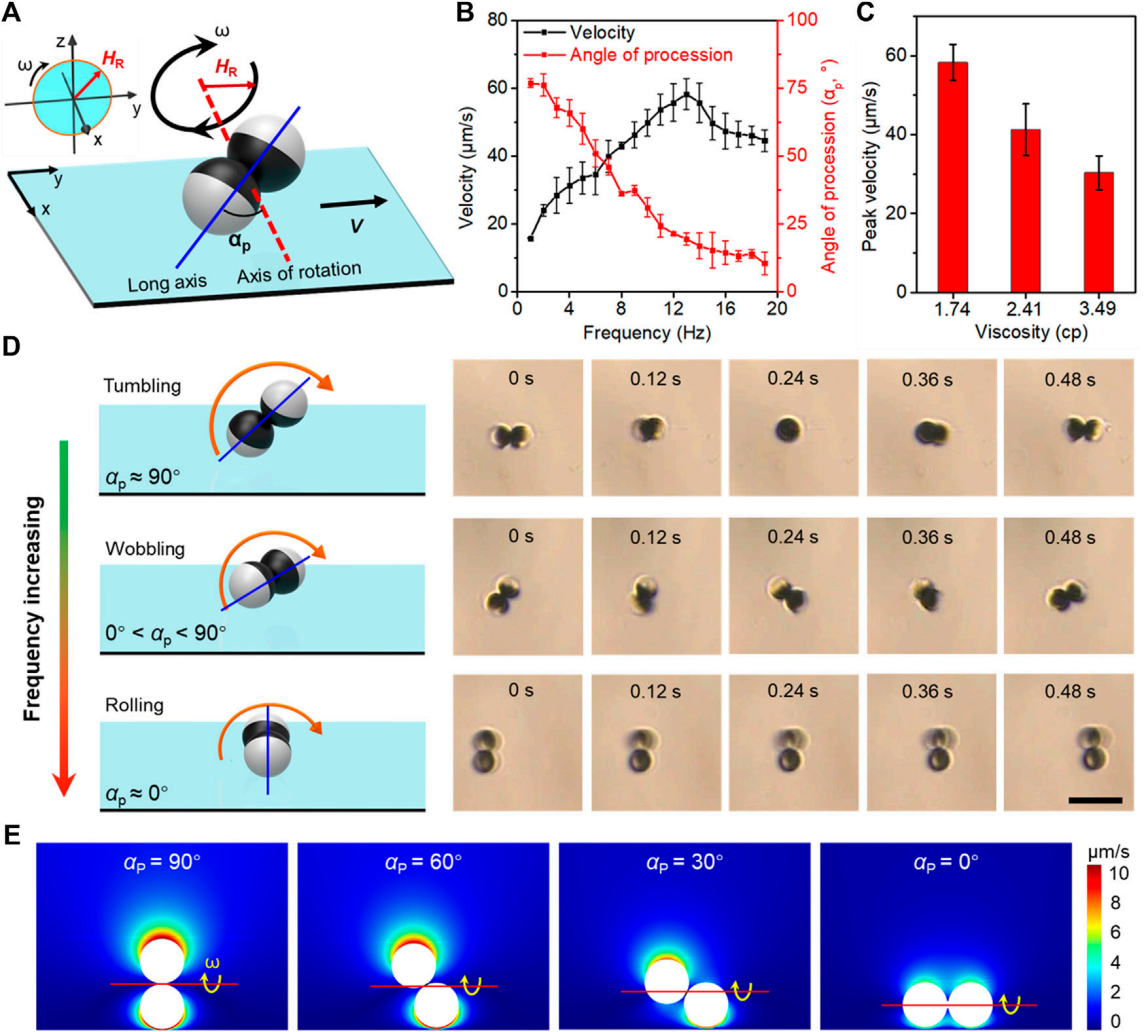
Actuation mechanisms of magnetic-propelled microdimers. **(A)** Schematic of a microdimer with permanent magnetization. The applied field is of strength *H*_R_ rotating at angular velocity *ω* (frequency f = *ω*/2π). **(B)** Velocity and angle of precession (*α*_p_) varied with the driving frequency in 10% glycerol–water solution (1.74 cp). **(C)** The peak velocity of microdimers in glycerol–water solution of different viscosities. **(D)** Schemes of microdimers in tumbling, rolling, and wobbling modes at different *α*_p_ under a rotating magnetic field. Scale bar 20 μm. **(E)** Simulation of the section view from one side of the microdimer shows the fluid flow velocity near a microdimer on the substrate at different *α*_p_.

Based on the axial symmetry and “peanut-like” shape of microdimer, the estimation of the concentration of the local samples from the dynamics of the microdimer can be concentrated on the locomotion velocity and posture (Lin et al., 2018). When the coercivity is much higher than the applied magnetic field strength, the magnetization strength and direction are considered unchanged. Figure 2A schematically illustrates the magnetization of microdimers under a rotating magnetic field. *H*_R_ and *ω* were magnetic field strength and angular velocity (related to frequency), respectively. The angle of precession (*α*_p_) is defined as the angle between the long axis and the axis of rotation. The long axis is defined as the connection between the centers of the two spheres of microdimers, and the axis of rotation is defined as the normal to the plane of rotating magnetic field. Excited by the stable input of the external magnetic field, the microdimer locomotes in a constant direction at a stable speed and posture in a homogeneous medium. The dynamics of the microdimer in 10% glycerol–water solution (∼1.74 cp) was imaged and analyzed to estimate *α*_p_, shown in Figure 2B. At a magnetic field strength of 5 mT, the velocity of microdimer increased linearly with the driving frequency and reached a maximum velocity of 55.8 μm s^−1^ (3.5 body length s^−1^) at 14 Hz, further increasing the frequency, which reduced the velocity. Such a maximum synchronized frequency is called step-out frequency, which occurred from the increase in drag caused by the increasing speed. In brief, the frequency corresponding to the peak speed of the microdimer is defined as step-out frequency. Simultaneously, frequency-induced increasing drag also forced the microdimer to change motion posture to weaken the fluid impact, expressing a decrease in *α*_p_. The *α*_p_ decreased from 76.8° to 4.2° upon increasing the driving frequency from 1 to 20 Hz. Figure 2C shows the peak velocity of microdimers in glycerol–water solutions of different viscosities. As can be seen, the peak velocity decreased with an increase in viscosity. Furthermore, the raised viscosity caused by the increasing glucose (or cholesterol and triglyceride) concentrations also enhances the resistance during locomotion, which further accelerated the occurrence of out-step frequency.

The microdimers exhibited three distributed locomotion modes, called tumbling, wobbling, and rolling (Figure 2D). When a uniform rotating magnetic field energizes them with increasing frequency, the essential details are as follows: 1) Under the excitation of the initial low-frequency magnetic field, the long axis of the microdimer was parallel to the rotation plane of the magnetic moment and perpendicular to the rotation axis, exhibiting a tumbling motion mode at *α*_p_ ≈ 90°. 2) As the frequency increases, the raised fluid drag compelled the long axis to deviate from the rotation plane of the magnetic field. At this time, the microdimer wobbling at 0° < *α*_p_ < 90° and can be divided into a rotation along its own axis and another homodromous rotation along an axis with an angle to the long axis. Step-out frequency also occurred during wobbling mode. 3) The wobbling mode gradually transformed into the rolling mode (*α*_p_ ≈ 0°) as out-of-step phenomenon intensified. The long axis of the microdimer almost coincides with the axis of rotation in the rolling mode. Note that the velocity and angle of precession in rolling was more stable than the tumbling and wobbling modes. The continuous motion process of the three modes at a magnetic field strength of 5 mT was captured from Supplementary Video S1 and is shown in Figure 2D.

The motion near the surface of the microdimer was impeded due to the hydrodynamic no-slip boundary, which created a mismatch of hydrodynamic interactions between the top and bottom and conversion of rotational motion to translational motion of microdimers. Microdimers will experience different drag forces in multimodes due to a discrepant fluidic interaction between them and the surrounding fluid. As shown in Figure 2E, the fluidic velocity field induced by a rotating microdimer at different precession angles (0°, 30°, 60°, and 90°) adjacent to the wall surface has been simulated and analyzed. All the simulations were performed within COMSOL Multiphysics 5.5 Simulation Software. For all motion mode groups, the total velocity acting on the microdimer increased with decreasing distance from the microdimer. Flow field strength at the same distance from the microdimer and the height of the rotation axis from the surface reduced with a decrease in the angle of precession.

### Estimation of Glucose Concentration

**FIGURE 3:**
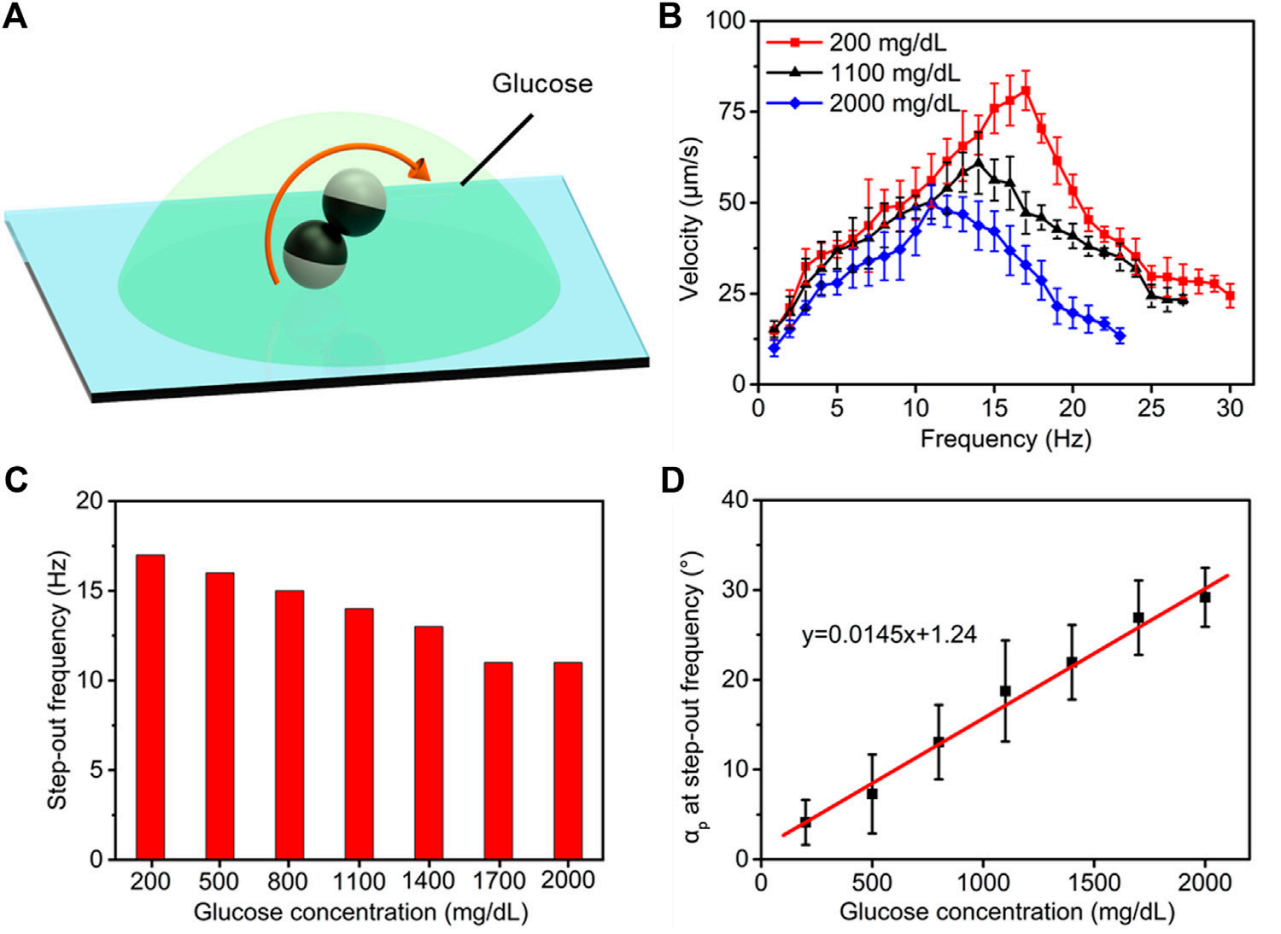
Estimating the strategy of plasma glucose levels. **(A)** Controllable motion of the microdimers in plasma with different glucose concentrations. **(B)** Variation in the velocity of the microdimers upon changing magnetic frequency and glucose concentration. **(C)** The step-out frequency at different glucose concentrations. **(D)** Function of the *α*_p_ at step-out frequency as increasing glucose concentration.

Point-of-care testing of plasma glucose and plasma lipids has been used to quickly diagnose diabetes mellitus (fasting glucose >126 mg/dl or glucose after 75 g of glucose oral load >200 mg/dl) and hyperlipidemia [total cholesterol (T-chol) >250 mg/dl and/or triglycerides (TG) >250 mg/dl]. In terms of the impact on the motility of microdimers, the concentration of glucose, cholesterol, and triglyceride will change the plasma viscosity and further affect their motion behavior. To overcome this limitation, we separately investigated the influence rules of the concentration of glucose, cholesterol, and triglycerides on the motility of microdimers to seek respective customized methods for the characterization of the three variables. Figure 3A schematically presents the locomotion of microdimers in plasma with preprogrammed glucose concentration. We executed the linear motion of the microdimer in plasma with different glucose concentrations and analyzed the relationship of the velocity and posture of the microdimers with the frequency of the applied magnetic field and glucose concentration. Figure 3B shows that under a rotating magnetic field of 5 mT and glucose concentration of 200 mg/dl, the velocity of the microdimer first went up linearly as the frequency grows, reaching a maximum velocity of 80.9 μm s^−1^ at a step-out frequency of 17 Hz. It then went down reciprocally after the step-out frequency. Interestingly, the motion displayed a series of peak velocities and step-out frequencies at different glucose concentrations, which decreased upon an increase in glucose concentration and dropped to 49.4 μm s^−1^ and 11 Hz when the glucose concentration was 2,000 mg/dl. A more detailed explanation about the rule of step-out frequencies and glucose concentration is presented in Figure 3C. Large-scale increase in glucose concentration did not significantly bring the occurrence of synchrony losing forward, which means that the change in glucose concentration processed a small effect on plasma viscosity. In addition, the procession angle of microdimers, dependent on both plasma viscosity and spinning velocity, also changed with the variational frequency and glucose concentration. The increase in frequency and glucose concentration both reduced the precession angle. In order to accurately estimate the glucose concentration, we measured the precession angle *α*_p_ as a function of step-out frequency as the glucose concentration increased after comparing the corresponding relationship between various parameters and glucose concentration (Figure 3D). The *α*_p_ at step-out frequency increased from 4.1° to 29.2° upon increasing the glucose concentration from 200 to 2,000 mg/dl. The linear fitting (red line) of the experimental data further reveals that the *α*_p_ at step-out frequency and the glucose concentration show a good linear relationship, which confirms the quantitative accuracy estimation of the glucose concentration using *α*_p_ at step-out frequency.

### Estimation of Cholesterol Level

**FIGURE 4:**
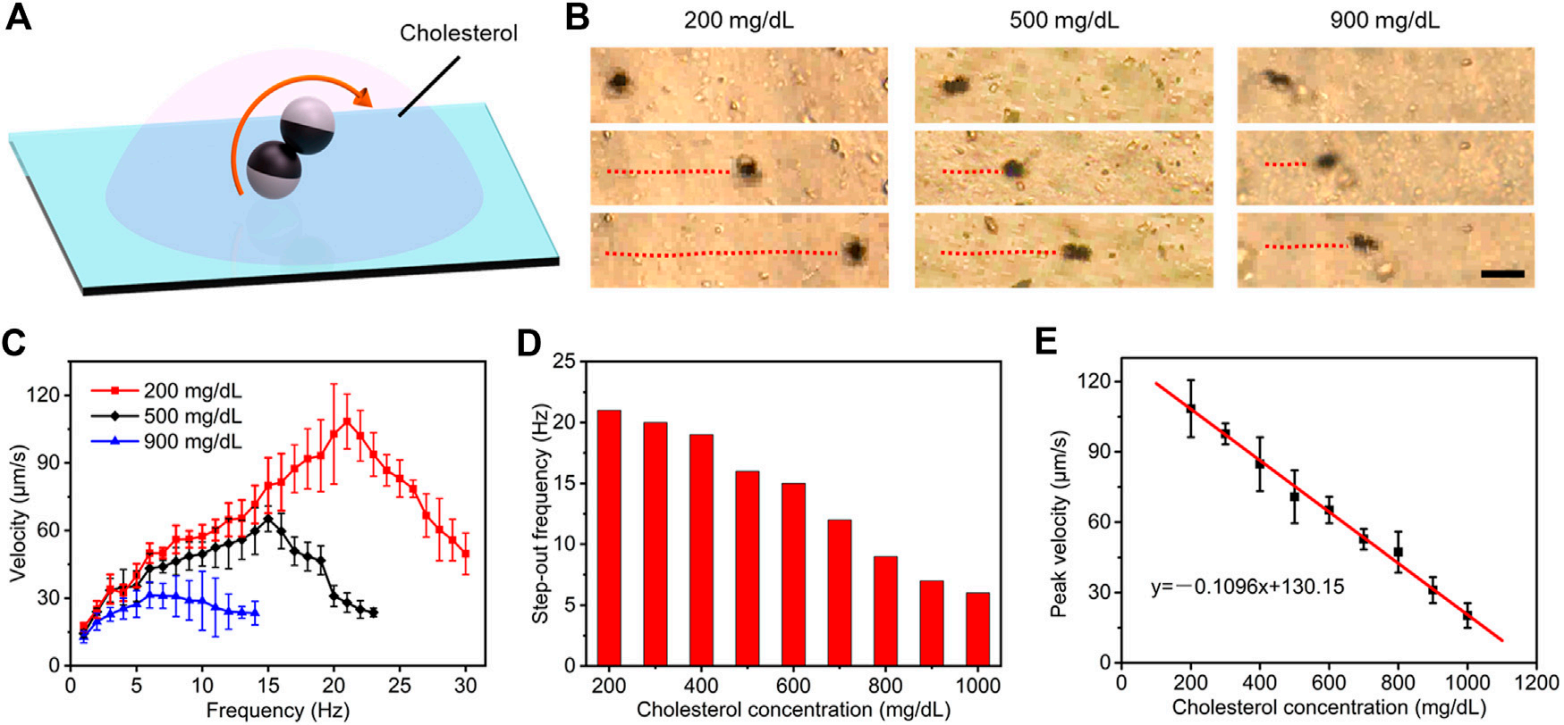
Estimating strategy of plasma cholesterol level. **(A)** Controllable motion of the microdimers in plasma with different cholesterol concentrations. **(B)** Time-lapse optical microscopy images of locomotion trajectories of microdimers propelled by rotating magnetic field in cholesterol concentrations of 200, 500, and 900 mg/dl. Scale bar: 20 μm. **(C)** Dependence of the translational speed of microdimer on the frequency of the external rotating magnetic and cholesterol concentration. **(D)** The step-out frequency at different cholesterol concentrations. **(E)** The peak velocity of microdimer was dependent on cholesterol concentration.

Cholesterol and triglycerides, as the primary targets of plasma lipid routine examination, are the main components that induce hyperlipidemia. Therefore, we explored specific estimation schemes for the concentration of cholesterol and triglycerides, respectively. Similar to the detection of plasma glucose, we perform microrobot operations in plasma with constant plasma glucose concentration and different plasma lipid concentrations, capturing its locomotion performance (Figure 4A). Time-lapse optical microscopy images in Figure 4B illustrates that locomotion trajectories and posture of microdimers propelled by rotating a magnetic field of 5 mT in cholesterol concentrations of 200, 500, and 900 mg/dl. Obviously, the velocity and procession angle of microdimers also were impacted evidently by the cholesterol concentration, and the former processed a stronger variation with the concentration. In view of this, we further analyzed the velocity of the microdimers changing with magnetic frequency under different cholesterol concentrations, as shown in Figure 4C. Compared with that in different glucose concentrations, the tendency of speed and the occurrence of step-out frequency were similar, but their decreasing amplitudes with an increase in cholesterol concentration were more significant. Particularly, when the concentration of cholesterol reached 900 mg/dl, the velocity increased from 12.8 μm s^−1^ at the minimum to only 31.4 μm s^−1^ at the maximum. This result indicates that increasing cholesterol concentration caused more apparent viscosity than glucose.

Next, we systematically investigated the step-out frequency and peak speed at different cholesterol concentrations. As described in Figure 4D, the step-out frequency decreased with an increase in concentration as nonlinear relationship, which limited the purpose of using step-out frequency to characterize cholesterol concentration. On the contrary, the peak velocity followed a linear relationship with the cholesterol concentration (Figure 4E). The linear fitting curve further illustrated that the estimation strategy based on peak velocity not only process a higher accuracy but also a large slope, which can be used to excellently estimate the concentration of cholesterol. A level of cholesterol over 250 mg/dl may indicate a greater risk for hyperlipidemia disease (Solá, et al., 2007). The experiments in this study cover the normal and abnormal cholesterol concentrations, so this strategy can be used to clinically estimate cholesterol level.

### Estimation of Triglyceride Level

**FIGURE 5:**
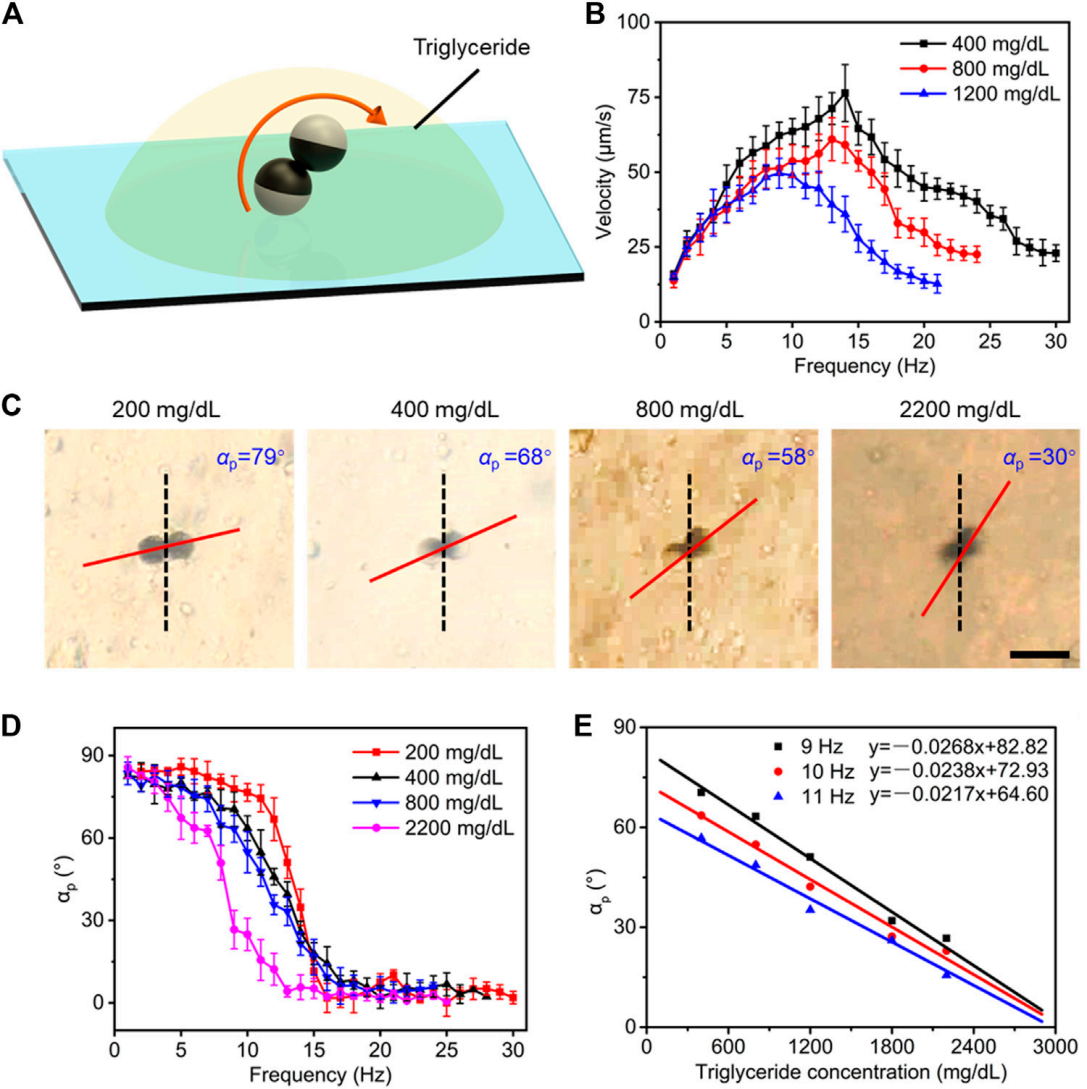
Estimating strategy of plasma triglyceride level. **(A)** Controllable motion of the microdimer in plasma with different triglyceride concentrations. **(B)** The velocity of microdimers varied with the drive frequency and triglyceride concentrations. **(C)** Snapshots of the moving microdimer and corresponding measured *α*_p_ at different triglyceride concentrations. Scale bar: 20 μm. **(D)** “Z” decreasing trend of precession angle with the increase in driving frequency. **(E)** Linear relationship of the *α*_p_ at 9, 10, and 11 Hz with triglyceride concentration.

Triglyceride level, as another lipoid component similar to cholesterol in plasma, also directly affects the plasma lipid level. The same strategy was used to seek an estimation method of triglyceride level (Figure 5A). The velocity of microdimers was also dependent on driving frequency and concentration of triglycerides, as shown in Figure 5B. Similarly, step-out phenomena asynchronously occurred in plasma with different triglyceride concentrations, and the greater concentration caused the lower step-out frequency. It also can be seen from Figure 5B that step-out frequency and maximum speed varied nonlinearly with triglyceride concentration. As a consequence, we turned to study the change in precession angle of microdimers to estimate the triglyceride concentration. A rotating magnet with strength of 5 mT and frequency of 10 Hz has a precession angle of microdimers at triglyceride concentrations of 200, 400, 800, and 2,200 mg/dl, respectively. The captured images are shown in Figure 5C. The *α*_p_ was 79° at a triglyceride concentration of 200 mg/dl and decreased with rising triglyceride concentration, which dropped to 30° at 2,200 mg/dl. We next measured the changing process of *α*_p_ with the frequency variating from 1 to 30 Hz. As described in Figure 5D, the precession angle represented a decreasing trend of “Z” shape with increasing frequency, which was the precession angle decreasing slightly in the low- and high-frequency bands, while the cliff-like decline occurred at the middle-frequency band. Interestingly, similar to the phase transformation of electrical signals, the cliff-like decline emerged earlier as the triglyceride concentration increased. This difference made the precession angle in middle middle-frequency band vary greatly with concentration change, which provided a guideline for estimating triglyceride concentration using *α*_p_. Similar changing trends of the precession angle were not observed in the case of plasma glucose or plasma cholesterol. The precession angle in the middle middle-frequency of 9, 10, and 11 Hz as a function of triglyceride concentration was selected and exhibited in Figure 5E. The excellent linear fitting results demonstrated the feasibility of estimating the triglyceride concentration based on the precession angle at the middle-frequency band.

## Conclusion

We first demonstrate that magnetic microdimer capable of dynamic motility performance with changing viscosity can be used to quantify the concentration of glucose, cholesterol, and triglyceride in plasma. The microdimers exhibited three locomotion modes of tumbling, wobbling and rolling with a change in resistance related to frequency and fluid viscosity, which were further supported by numerical studies. These results show the superiority of using precession angle *α*_p_ at step-out frequency and peak speed to estimate glucose and cholesterol levels, respectively. As for the measurement of triglyceride level, there is a linking synchronization in *α*_p_ changing the trend with variating frequency and triglyceride concentration. The dynamic ranges in concentration measurements of glucose, cholesterol, and triglyceride were almost 2,000, 1,000, and 3,000 mg/dl, respectively. This technique was envisaged to apply for detection in more scenarios that require fast mechanical changes.

## Data Availability

The raw data supporting the conclusion of this article will be made available by the authors, without undue reservation.
